# Temporal Dynamics of Fecal Microbiome and Short-Chain Fatty Acids in Sows from Early Pregnancy to Weaning

**DOI:** 10.3390/ani15152209

**Published:** 2025-07-27

**Authors:** Sui Liufu, Xin Xu, Qun Lan, Bohe Chen, Kaiming Wang, Lanlin Xiao, Wenwu Chen, Wu Wen, Caihong Liu, Lei Yi, Jingwen Liu, Xianchuang Fu, Haiming Ma

**Affiliations:** 1 College of Animal Science and Technology, Hunan Agricultural University, Changsha 410128, China; 1304321378@stu.hunau.edu.cn (S.L.); xuxin141596@163.com (X.X.); lanqunstrive@163.com (Q.L.); chenhe0213914@163.com (B.C.); 15116529648@163.com (K.W.); finafantacyla@163.com (L.X.); cww1242646778@163.com (W.C.); ww15979545900@163.com (W.W.); 17679179352@163.com (C.L.); ylasoi@163.com (L.Y.); 15093946542@163.com (J.L.); fuxc0313@163.com (X.F.); 2 Yuelushan Laboratory, Changsha 410128, China; 3 Key Laboratory of Livestock and Poultry Resources Evaluation and Utilization, Ministry of Agriculture and Rural Affairs, Changsha 410128, China

**Keywords:** sow, pregnancy to weaning, feces, 16S rRNA gene sequencing, SCFAs

## Abstract

Although age-related changes in the gut microbiome of pigs have been extensively studied, the dynamic patterns of fecal microbiota and SCFAs during the gestation-to-weaning period in sows remain poorly characterized. We aim to characterize the changes in fecal microbiota and SCFAs from pregnancy to weaning, and to investigate their associations with maternal weight gain during gestation. We systematically collected 100 fecal samples at four time points (day 30 of pregnancy (T1), 1–2 days before delivery (T2), day 10 after delivery (T3), and day 21 of weaning stage (T3)), and measured the body weight of sows at T1 (132 kg ± 10.8) and T2 (205 kg ± 12.1). The primary nutrient components of the diets during the gestation and lactation periods are summarized. All fecal samples were subjected to 16S rRNA gene sequencing. We found that a high proportion of crude fiber (bran) is a key feature of the gestation diet, which may affect enterotype shifts and gut microbial composition. Sows fed a high-fiber diet showed significant enrichment of gut microbiota, including genera such as *Prevotellaceae_UCG-003*, *Prevotellaceae_NK3B31_group*, and *Prevotella_9* during the gestational period (LDA score > 2). Moreover, *Eubacterium_coprostanoligenes_group* (average relative abundance: 5.5%) and *Lachnospiraceae_NK4A136_group* (average relative abundance: 2.5%) were the dominant bacteria during the lactation stage. Fecal propionate and butyrate levels were lowest in late gestation, and propionate negatively and acetate positively correlated with body weight change (*p* < 0.05). Additionally, certain *Prevotella* taxa were associated with arachidonic acid metabolism and acetate production (*p* < 0.05). Our study identified key microbial communities across four stages from gestation to weaning and revealed that dietary patterns can shape the sow gut microbiota. Furthermore, we observed significant correlations between SCFAs and body weight change during pregnancy. These findings provide a scientific basis and theoretical support for future strategies aimed at modulating gut microbiota and targeting SCFAs to improve maternal health and productivity throughout the gestation-to-weaning period.

## Simple Summary

The gut microbiota and its metabolites, particularly short-chain fatty acids (SCFAs), play crucial roles in maternal metabolism and health during pregnancy. However, the dynamic changes in fecal microbiota and SCFAs across the entire reproductive cycle—from pregnancy to weaning—in sows remain poorly understood. In this study, we systematically characterized the gut microbial composition and SCFA profiles of 25 sows at four key time points: early pregnancy (T1), late pregnancy (T2), early lactation (T3), and weaning (T4). We found that high-fiber diets during gestation may influence the composition of the gut microbiota. During pregnancy, *Prevotella* species are the predominant microbes in the sow gut, while *Eubacterium_coprostanoligenes_group* and *Lachnospiraceae_NK4A136_group* became dominant in the microbiota during lactation. Notably, fecal propionate levels were negatively correlated with body weight change, while acetate showed a positive correlation. Additionally, certain *Prevotella* species were linked to arachidonic acid metabolism and SCFA production. These findings highlight the importance of the gut microbiota and SCFAs during the gestation-to-weaning period in sows, and provide a scientific basis for future microbiome-targeted interventions aimed at improving maternal health.

## 1. Introduction

The symbiotic relationship between the host and microbiota plays a crucial role throughout the entire life cycle. Notably, dynamic changes in maternal microbial communities profoundly affect host and offspring health [1,2]. In recent years, there has been growing interest in elucidating the developmental patterns of mammalian gut microbiota and their interplay with host physiological conditions [3]. During pregnancy, the dramatic shifts in host hormone levels, metabolic demands, and immune responses are associated with structural changes in the gut microbiota [2,4,5]. In females, the structure and function of intestinal microbial communities may experience systematic reorganization during the transition from pregnancy to lactation. However, most existing studies have focused on microbial characteristics within specific developmental windows, lacking comprehensive analyses that span the early pregnancy through the end of lactation [6]. This limitation hinders a holistic understanding of the mechanisms underlying microbe–host interactions.

The domestic pig (*Sus scrofa*) plays a crucial role in global agriculture and is responsible for more than 40% of total meat output worldwide [7]. Animal breeding is a highly profitable industry and is considered an important means of improving national economies [8]. The performance of sows during the gestation-to-weaning period is a key focus for breeders, as this stage is closely linked to their productivity. In recent years, alongside advancements in breeding techniques, the gut microbiome has received widespread attention as the so-called “second genome” [9]. Microbial-produced metabolites play a vital role in modulating maternal metabolic homeostasis [10]. The gut contains high millimolar (mM) concentrations of SCFAs, which are generated by gut bacteria through the anaerobic fermentation of soluble fiber [11]. During the course of pregnancy, SCFAs are absorbed into the maternal circulation and subsequently transported across the placental barrier, where they play a role in supporting fetal organ development and growth [12]. Researchers indicated that elevated maternal serum SCFA levels have been linked to positive effects on weight gain, glucose metabolism, and the regulation of various metabolic hormones [13]. Serum acetic acid levels were found to be positively associated with maternal weight gain and adiponectin concentrations, whereas serum propionate levels exhibited a negative correlation with maternal leptin levels [13]. Adipokines, such as adiponectin and leptin, are hormones secreted by adipocytes and play a critical role in metabolic adaptations during pregnancy. These hormones contribute to the regulation of satiety, insulin sensitivity, and the development of obesity [14]. A recent study revealed that maternal levels of propionic acid tend to decline as pregnancy progresses, whereas its levels were elevated in obese pregnant women [15]. Another investigation in rats revealed that pregnancy is accompanied by elevated levels of acetic and propionic acid, along with a potential increase in butyric and straight-chain hexanoic acid concentrations [16]. These findings from previous studies suggest that the changes in SCFAs during pregnancy may be influenced by genetic background and other physiological or environmental factors.

Human studies often face challenges related to time span, environmental control, and ethical restrictions. In this context, pigs provide distinct advantages as a monogastric animal model because of their remarkable similarities to humans in terms of intestinal structure and fat deposition [17], rendering them highly suitable for investigating host–microbiome interactions. In addition, the gut microbiome is a critical component of human and animal metabolism and overall health. The composition of the maternal gut microbiome contributes to obstetric outcomes and has long-term health implications for both the mother and her offspring. In intensive swine farming, the gut health of sows is crucial for the well-being of their offspring and may ultimately influence the overall economic performance of the farm. In this study, we collected fecal samples from 25 sows at four time points, ranging from early pregnancy to the weaning stage, and the fecal concentrations of SCFAs were also determined. Our study focuses on the following critical issues: (1) the main enterotypes across the four critical stages from pregnancy to lactation; (2) the core gut microbes during these four critical stages; (3) the dynamic changes in fecal SCFAs from pregnancy to weaning; (4) exploration of the associations among microbial taxa, SCFAs, and body weight change during pregnancy. The relatively large sample size of sows in this study represents a major advantage, contributing to a more representative and statistically robust analysis. Moreover, this study, for the first time, characterized the dynamic changes in SCFAs from pregnancy to weaning in sows. We hypothesize that the unique gut microbial composition and SCFA levels observed across the gestation and postpartum periods may reflect underlying shifts in overall maternal metabolism, with potential implications for maternal pregnancy and offspring developmental outcomes. Our findings are intended to support the development of microbiome- and metabolite-based interventions for improving sow pregnancy outcomes and postpartum health, offering potential insights and a theoretical foundation for research in human pregnancy.

## 2. Materials and Methods

### 2.1. Experimental Animals and Sampling

The animals and experimental protocols used in this study followed the guidelines of the Ministry of Agriculture of China and the Committee of Animal Care at Hunan Agricultural University (Changsha, China) (Permit Number: CACAHU 20230630; Approval Date: 30 June 2023). To investigate the dynamic changes in the fecal microbiota of sows from pregnancy to weaning, we selected 25 Yorkshire sows (average age: 220 days ± 5.5; body weight: 132 kg ± 10.8) for fecal sample collection. All sows were housed in a large-scale pig farm in Fujian Province, China, under the same environmental and dietary conditions. The primary nutrient components of the diets are enumerated in Table S1. A total of 100 fecal samples were obtained from four key stages: (T1) day 30 of pregnancy, (T2) 1–2 days before delivery, (T3) day 10 after delivery, (T4) weaning stage (day 21). All stool samples were stored in 2 mL sterilized plastic sampling tubes and then immersed in liquid nitrogen for future DNA extraction and SCFA determination. All experimental pigs were healthy and had not been fed with probiotics or drugs within at least 2 months. Moreover, we measured the body weights of sows at the T1 and T2 stages to calculate the body weight change during the pregnancy stage. Considering the effects of age and parity on body weight, we used a residual-based linear model to correct the phenotype (body weight) and used it to calculate the body weight change. The formula was as follows: body weight change = [(corrected body weight at the T2 stage) − (corrected body weight at the T1 stage)/pregnancy days].

### 2.2. Bacterial DNA Isolation, 16S rRNA Gene Sequencing, and Bioinformatics

DNA extraction was conducted using the QIAamp^®^ stool mini kit (QIAGEN, Hilden, Germany) according to the operating protocols. All subsequent steps adhered to the operating manual, except for the final step, where the columns were eluted using 30 μL of diethyl pyrocarbonate (DEPC)-treated water. The DNA solution was quantified with NanoDrop One (Thermo Fisher Scientific, Waltham, MA, USA) and stored at −80 °C for follow-up sequencing. Then, microbial DNA amplification was carried out using the primer pair 338F (5′-ACTCCTACGGGAGGCAGCA-3′) and 806R (5′-GGACTACHVGGGTWTCTAAT-3′), targeting the V3-V4 region of the 16S rRNA gene. Both of the primers were added with Illumina sequencing adapters. The PCR amplification steps were as detailed below: initial denaturation (95 °C for 5 min), primer annealing (25 cycles of 95 °C 30 s, 50 °C 30 s, and 72 °C 40 s), and a final extension step for 7 min at 72 °C. Amplicon sequencing was performed on a MiSeq platform (Illumina, San Diego, CA, USA) according to the standard paired-end instructions (2 × 250 bp).

Raw sequence data, including barcode and primer information, were first removed using Cutadapt (v4.7). Using DADA2, paired-end sequences were denoised, and chimeras were also discarded (method = “consensus”). The representative sequences for each Amplicon Sequence Variant (ASV) were annotated to taxonomies using DADA2 with a naive Bayesian classifier against the Silva v138 (version 2) database. The above steps were performed in Quantitative Insights Into Microbial Ecology (QIIME, v1.80) software. Alpha diversity was assessed using the Shannon index, while beta diversity was measured with unweighted UniFrac distances, visualized through principal coordinates analysis (PCoA). ASVs at the genus level that appeared in at least 20% of individuals were retained. Permutational multivariate analysis of variance (PERMANOVA) based on the microbial dataset was performed to detect significant differences among different stages. The ‘vegan’ package in the R environment (v4.2.3) was used for statistical analysis.

### 2.3. Enterotype Analysis

Gut microbial enterotypes were identified through an integrated computational approach. Samples were clustered using the Jensen–Shannon Divergence (JSD) metric and Partitioning Around Medoids (PAM) algorithm [18], based on genus-level relative abundance profiles. The optimal number of clusters was determined by maximizing the Calinski–Harabasz (CH) index [19], which evaluates the ratio of between-cluster to within-cluster variance. To ensure statistical robustness, the silhouette coefficient of the observed clusters was compared against a null distribution generated from simulated datasets. This pipeline combines rigorous statistical validation with robust clustering to delineate distinct enterotypes.

### 2.4. Biomarker Discovery Using 16S rRNA Dataset

To identify the microbial biomarkers and significantly enriched KEGG pathways at each stage, linear discriminant analysis effect size (LEfSe) was performed using a normalized ASV relative abundance matrix. As a powerful tool for high-dimensional biomarker discovery, the LEfSe algorithm employs the non-parametric Kruskal–Wallis test to detect features with significant differences among groups, and it performs linear discriminant analysis (LDA) to estimate the effect size of each feature. In this study, the LDA score threshold and significance level were set at 2 and 0.05, respectively.

### 2.5. Cluster Pattern of Gut Microbial Profiles

Fuzzy clustering of gut microbial abundance data (top 500) was performed using the Mfuzz package (v3.62) in the R environment [20]. Prior to analysis, microbial abundance profiles (at the genus level) were normalized using variance-stabilizing transformation (VST) to mitigate compositional bias. The optimal number of clusters was determined by evaluating the minimum centroid distance and cluster validity indices (e.g., silhouette coefficient). Fuzzy c-means clustering was applied, with the c (number of clusters) and m (fuzzification coefficient) parameters optimized through iterative grid search. To assess statistical robustness, cluster stability was validated via bootstrapping (1000 iterations), and significant clusters were defined as those with membership scores > 0.7 and adjusted *p*-values < 0.05 (Benjamini–Hochberg correction). Cluster centroids and membership probabilities were visualized using heatmaps and 3D ordination plots to depict microbial co-abundance patterns.

### 2.6. Determination of Fecal SCFAs

To visualize the correlations among different gut microbial taxa, we constructed a correlation network using Cytoscape (v3.7.2). First, pairwise Spearman correlation coefficients were calculated between the relative abundances of ASVs (top 500) based on their normalized abundance profiles. ASVs were retained for network construction based on a predefined threshold at |r| > 0.7 and FDR < 0.001. The correlation matrix was then exported in a tab-delimited edge list format, where each row represented a pair of nodes (microbial taxa) and their associated correlation value. This edge list was imported into Cytoscape, and a network graph was generated, with nodes representing microbial taxa and edges representing significant correlations (positive or negative).

### 2.7. Statistical Analysis

All statistical analyses were performed in the R environment (v4.2.3). The Shannon index was used to compare the α-diversity among the four groups. The β-diversity was assessed through principal coordinates analysis (PCoA) using Bray–Curtis distances, implemented with the “vegan” package [21]. We performed a multiple-stage comparison of fecal SCFAs using one-way ANOVA to evaluate stage-specific differences. The normality of distribution for body weight change and SCFA levels was assessed using the Shapiro–Wilk test. Spearman’s rank correlation was employed to evaluate the associations between body weight change and SCFA concentrations. Analysis of functional pathway was performed using PICRUSt based on the Kyoto Encyclopedia of Genes and Genomes (KEGG) database. The *p*-values from the correlation analysis among ASVs were adjusted for multiple testing using the Benjamini–Hochberg procedure (FDR correction). Results with a *p*-value <0.05 were considered statistically significant.

## 3. Results

### 3.1. Temporal Dynamics of Fecal Bacterial Community from Pregnancy to Weaning

**Figure 1:**
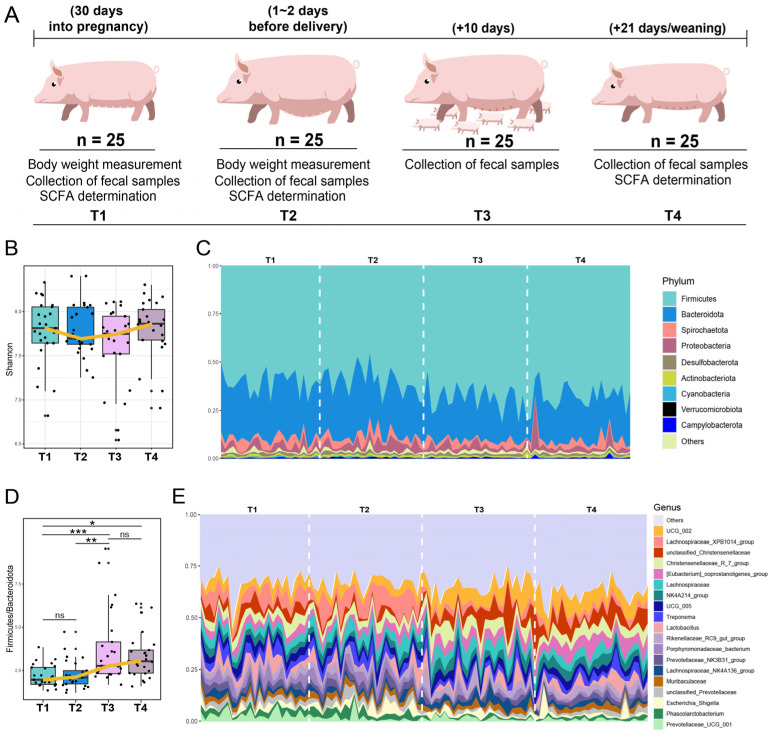
Longitudinal analysis of sow gut microbiota from pregnancy to weaning. (**A**) Schematic representation of study design. Sows (n = 25) sampled at four different time points, including when pregnant for 30 days (T1), 1–2 days before delivery (T2), at postnatal day 10 (T3), and in weaning stage (day 21) (T4). (**B**) Comparison of Shannon index among four stages. (**C**). Gut microbial composition of sows at phylum level from T1 to T4. (**D**) Comparison of Firmicutes/Bacteroidota (F/B) ratio among four stages. (**E**) Gut microbial composition of sows at genus level from T1 to T4. * *p* < 0.05, ** *p* < 0.01, *** *p* < 0.001; ns: no significant difference.

Fecal samples were obtained sequentially from 25 female pigs across four key stages: (T1) day 30 of pregnancy, (T2) 1–2 days before delivery, (T3) day 10 after delivery, (T4) weaning stage (day 21) (Figure 1A). A total of 9.01 million reads (average: 90.13 K; range: 73.36–142.62 K) were generated from the 100 samples (Table S2). The average raw read counts varied significantly across different age points, with samples collected at the T1, T2, and T3 stages exhibiting significantly lower read counts than those collected at the weaning stage (T4) (Figure S1, *p*  <  0.05). Following the removal of low-quality reads using DADA2, a total of 8.09 million high-quality sequencing reads were retained. Finally, a total of 2409 ASVs were included in the downstream analysis.

The Shannon index showed no significant differences across the four stages, while we found that the lowest level of the Shannon index was observed at the T2 stage, with a subsequent progressive increase after piglet delivery (Figure 1B, Table S3). In addition, we found that the proportion of Firmicutes increased from T1 (mean 60.4%) to T4 (mean 67.4%), while the proportion of Bacteroidota decreased from T1 (mean 28.8%) to T4 (mean 22.6%) (Figure 1C). After comparing the Firmicutes to Bacteroidota (F/B) ratio across the four stages, we found that the F/B values were significantly higher at T3 and T4 compared to T1 (*p* < 0.05, Figure 1D). Moreover, the overall trend of F/B value showed an increase over time. At the genus level, the bacteria *UCG_002*, *Lachnospiraceae_XPB1014_group*, *unclassified_Christensenellaceae*, *Christensenellaceae_R-7_group*, and *Eubacterium_coprostanoligenes_group* were ranked as the top five (Figure 1E). Particularly, the relative abundances of four genera, including *UCG_002*, *unclassified_Christensenellaceae*, *Christensenellaceae_R-7_group*, and *Eubacterium_coprostanoligenes_group*, showed a progressive increase after delivery. In addition, a high proportion of bran is a key feature of the gestation diet, while the lactation diet is primarily characterized by a high content of soybean meal and corn (Table S1).

### 3.2. Enterotype Changes from Gestation to Weaning

**Figure 2:**
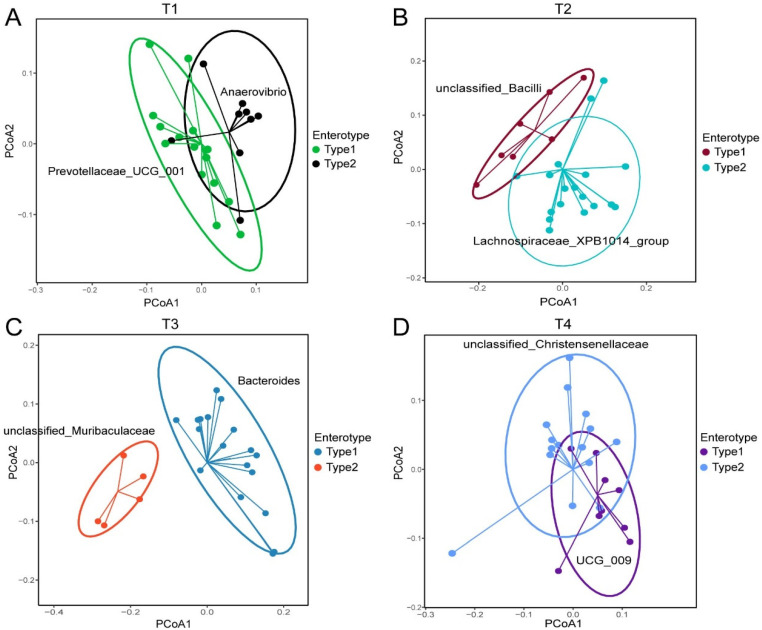
The fecal bacterial enterotype transition in sows from early pregnancy to weaning. (**A**–**D**) Two distinct enterotypes were identified in the T1 (**A**), T2 (**B**), T3 (**C**), and T4 (**D**) stages.

Enterotype analysis enables the identification of distinct microbial community structures and their associations with host health. The optimal number of enterotypes at each stage was determined based on the Calinski–Harabasz (CH) index (Figure S2). The microbial communities of the sows at T1 were dominated by two enterotypes (*Prevotellaceae_UCG_001* and *Anaerovibrio*) (Figure 2A). As the pigs progressed to T2, the enterotype shifted to another two ASVs, namely *Lachnospiraceae_XPB1014_group* and *Unclassified_bacilli* (Figure 2B). Ten days after delivery, we found that *Bacteroides* and *unclassified_ Muribaculaceae* were the dominant enterotypes (Figure 2C). At the time of litter weaning, the enterotype shifted to *unclassified_Christensenellaceae* and *UCG_009* (Figure 2D).

### 3.3. Identification of Gut Microbial Biomarkers in Sows from Gestation to Weaning

**Figure 3:**
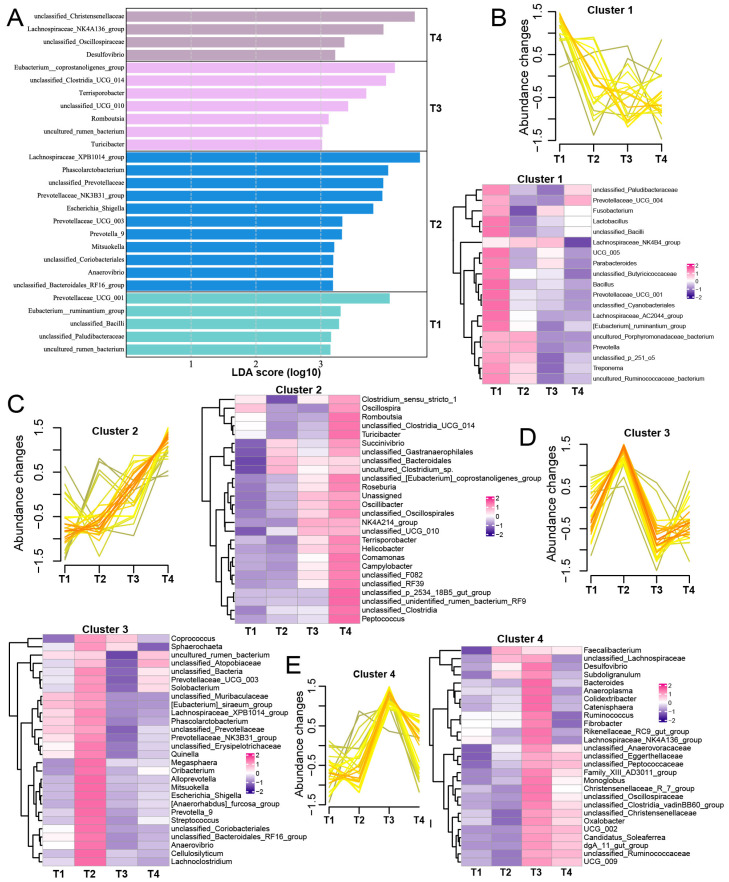
The identification of microbial biomarkers and abundance change patterns among the four stages. (**A**) The identification of significantly different ASVs among the four stages using LEfSe analysis; LDA score > 2. (**B**–**E**) The clustering patterns and abundance differences in each cluster, including cluster 1 (**B**) with a gradually decreasing abundance, cluster 2 (**C**) with a gradually increasing abundance, cluster 3 (**D**) showing elevated abundance at the T2 stage, and cluster 4 (**E**) displaying increased abundance at the T3 stage.

We used LEfSe analysis to further identify microbial biomarkers across different stages (Figure 3A). The genera *Prevotellaceae_UCG_001* and *Eubacterium_ruminantium_group* were specifically found in T1. For the T2 stage, the microbial biomarkers included *Lachnospiraceae_XPB1014_group*, *Phascolarctobacterium*, and four ASVs belonging to *Prevotellaceae,* including *Prevotellaceae_NK3B31_group*, *Prevotellaceae_UCG_003*, *unclassified_Prevotellaceae*, and *Prevotella_9*. Ten days after delivery, we found that *Eubacterium_coprostanoligenes_group*, *unclassified_Clostridia_UCG_014*, and *Terrisporobacter* were the dominant bacteria, while for the T4 stage, we found that *unclassified_Christensenellaceae* and *Lachnospiraceae_NK4A136_group* were highly enriched.

Next, we used the R package “Mfuzz” to analyze the trends of gut microbiota based on the relative abundance of ASVs (top 100). We found that *Lactobacillus*, *Bacillus*, *unclassified_Cyanobacteriales*, *Lachnospiraceae_AC2044_group*, and *UCG_005* in cluster 1 showed a gradual decrease in abundance with time (Figure 3B). Moreover, bacteria, including *Turicibacter*, *Romboutsia*, *Roseburia*, and *Peptococcus* in cluster 2 exhibited a progressively increasing abundance (Figure 3C). In cluster 3, *Cellulosilyticum*, *Lachnoclostridium*, *Streptococcus*, and *Escherichia_Shigella* showed upregulated abundance at the T2 stage (Figure 3D). Finally, we found that cluster 4 was mainly characterized by ASVs at the T3 stage (e.g., *Colidextribacter* and *Catenisphaera*) (Figure 3E).

### 3.4. Gut Microbial Interaction Networks

**Figure 4:**
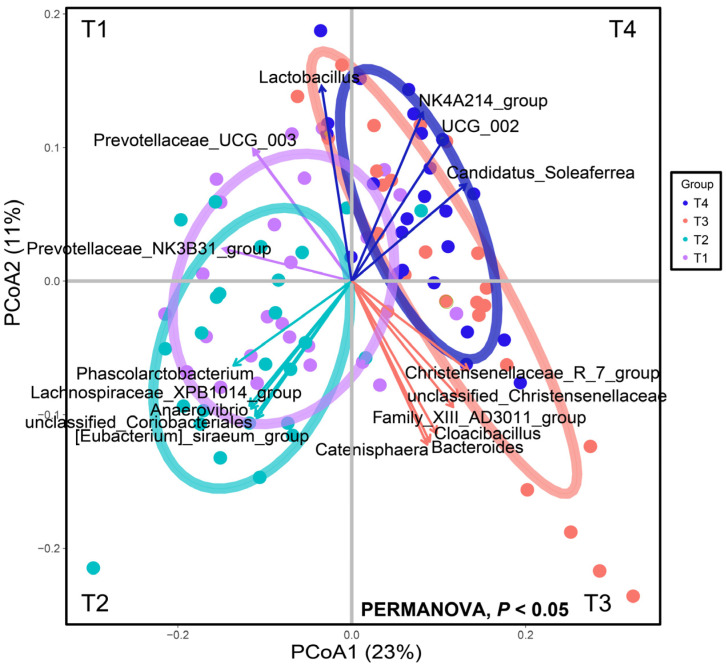
Principal coordinates analysis of gut microbial composition based on Bray–Curtis distances among four stages. Bacterial species that significantly correlated with principal components (PCs) with correlation coefficients > +0.7 or <−7 (Spearman’s correlation coefficient) plotted as contributors that drive separation. Length of arrow indicates degree of correlation with PCs.

First, we found significant differences in the clustering of gut microbiota across the four stages (PERMANOVA, *p* < 0.05, Figure 4). Similarly to the LEfSe results, we found that ASVs belonging to *Prevotellaceae* contributed greatly to microbial profiles of the T1 stage, while microbial profiles of the T3 stage were driven by *Family_Xll_AD3011_group*, *Catenisphaera*, and *Cloacibacillus*, etc., ASVs, including *NK4A214_group*, *UCG_002*, *Candidatus_Soleaferrea*, which were the “driver” bacteria in the T4 stage.

**Figure 5:**
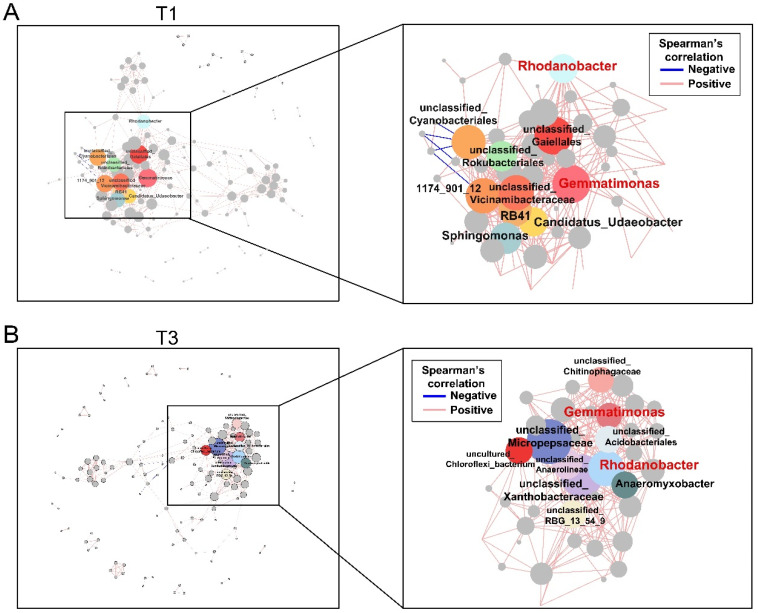
Interactions among the bacteria. (**A**) The microbiota interaction networks were analyzed at the T1 (**A**) and T3 (**B**) stages based on the following criteria: |r| > 0.7 and FDR < 0.001. The pink and blue lines indicate positive and negative correlations, respectively.

Next, we aimed to explore the interaction among different bacteria via performing a correlation analysis using the ASVs (top 500) in each stage (|r| > 0.7 and *p* < 0.001). In the T1 stage, we found that several bacteria possessed higher degrees of positive correlation, including *Candidatus_udaeobacter*, *Gemmatimonas*, *Sphingomonas*, and *Rhodanobacter* (Figure 5A). In the T2 stage, *Candidatus_udaeobacter*, *Flavobacterium*, *Mucispirillum*, and *Vicinamibacteraceae* were positively associated with each other and displayed higher degrees of correlation (Figure S3A). Regarding the T3 stage, we observed that two genera, *Rhodanobacter* and *Gemmatimonas*, exhibited a higher degree of correlation, a pattern also observed in the T1 stage (Figure 5B). This suggests that these two ASVs may play important roles during the postpartum recovery period. At the T4 stage, we noticed that *Prevotellaceae_Ga6A1_group*, *Hydrocarboniphaga*, *Taibaiella*, *Mesorhizobium*, and *Sphingobium* were positively associated with each other (Figure S3B).

### 3.5. The Functional Characteristics of Gut Microbiota

**Figure 6:**
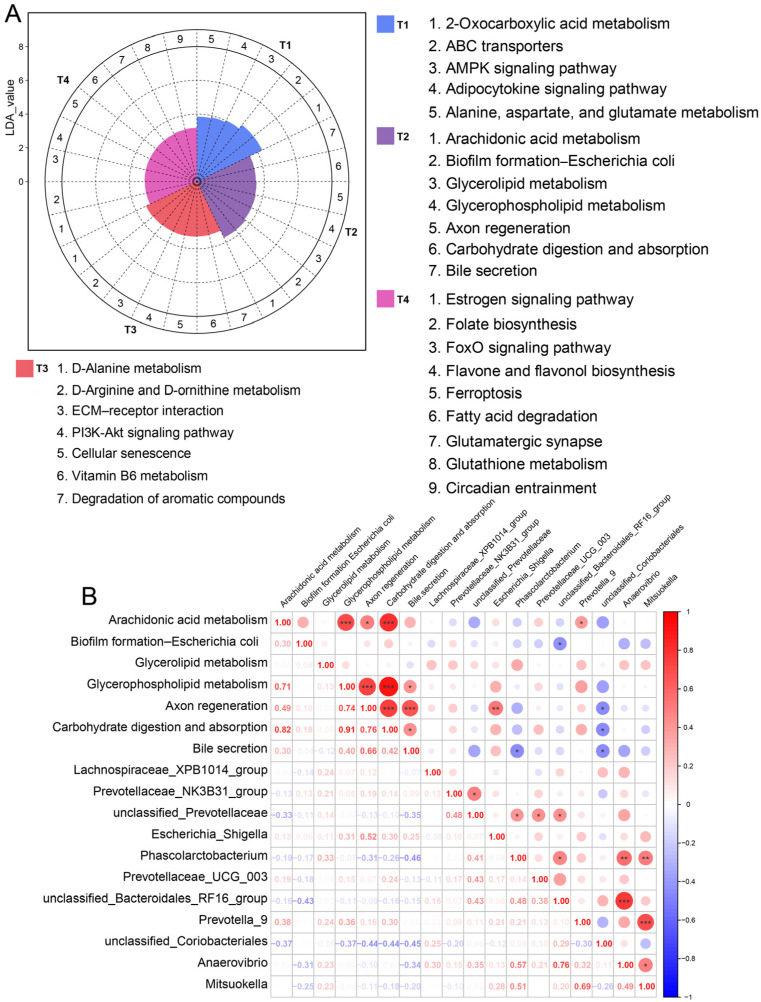
Differences in bacterial function and their associations with gut microbiota. (**A**) The identification of differentially enriched KEGG pathways across the four stages. (**B**) The correlation analysis between the differentially enriched KEGG pathways and gut microbial biomarkers identified at the T2 stage. Spearman’s rank correlation analysis. * *p* < 0.05, ** *p* < 0.01, *** *p* < 0.001.

PICRUSt was used to predict KEGG pathways of gut bacterial communities. Here, a total of 28 differentially enriched KEGG pathways were detected in the sow gut microbiota among the four stages (Figure 6A). We identified that ABC transporters, the AMPK signaling pathway, and the adipocytokine signaling pathway were more active in the sow gut microbiome at the T1 stage. Simultaneously, at the T2 stage, the gut microbiota was more capable of arachidonic acid metabolism, glycerolipid metabolism, and carbohydrate digestion and absorption. At the T3 stage, it was mainly enriched in the pathways of ECM–receptor interaction, vitamin B6 metabolism, and degradation of aromatic compounds. For the T4 stage, we found that the estrogen signaling pathway, fatty acid degradation, glutamatergic synapse, and glutathione metabolism were more active. Next, we performed an association analysis between gut microbiota and KEGG functions at the T2 stage. We found that *Prevotella_9* was positively associated with arachidonic acid metabolism (Figure 6B) and *Phascolarctobacterium* was negatively associated with bile secretion.

### 3.6. Association Among Gut Microbiota, SCFAs, and Body Weight Change During Pregnancy

**Figure 7:**
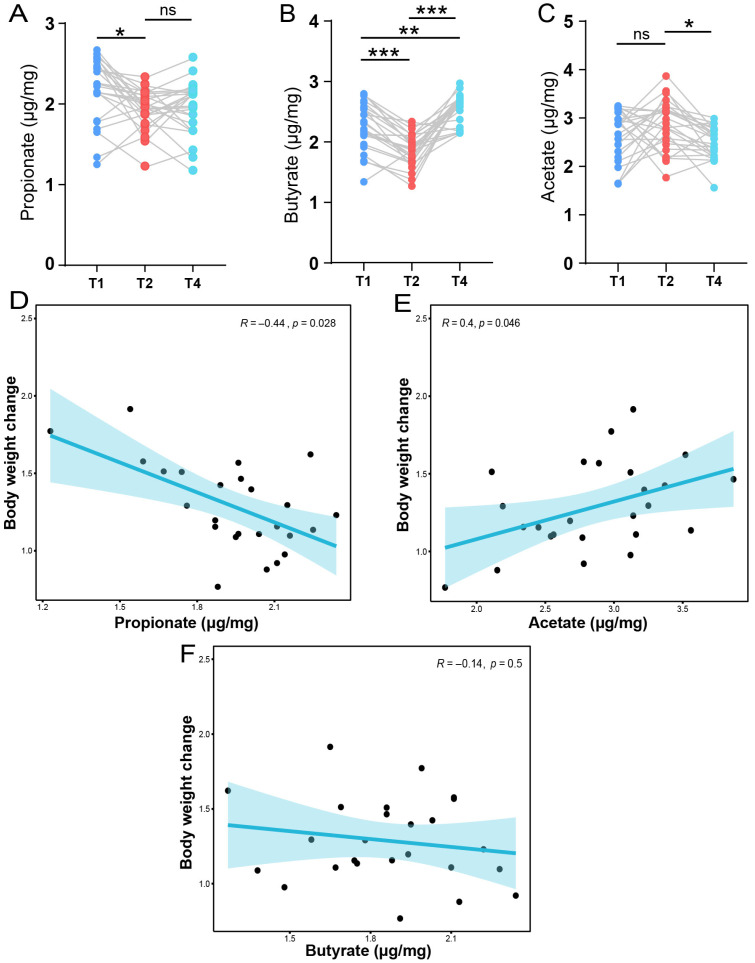
The dynamic changes in SCFAs and their correlation with body weight change during gestation. The comparison of fecal propionate (**A**), butyrate (**B**), and acetate (**C**) among the three main stages from pregnancy to weaning—one-way ANOVA. (**D**) The correlation analysis between fecal propionate and body weight change. (**E**) The correlation analysis between fecal acetate and body weight change. (**F**) The correlation analysis between fecal butyrate and body weight change. Spearman’s rank correlation analysis. * *p* < 0.05, ** *p* < 0.01, *** *p* < 0.001; ns: no significant difference.

By analyzing the levels of fecal SCFAs, we observed significant variations in the levels of acetate, propionate, and butyrate across different stages. Specifically, fecal propionate and butyrate levels decreased progressively from the T1 to the T2 stage, followed by an increase at the T4 stage (Figure 7A,B). Additionally, we observed significant differences in fecal propionate and butyrate levels between the T1 and T2 stages (*p* < 0.05). Interestingly, we observed that acetate levels were highest at the T2 stage (Figure 7C), while butyrate levels peaked at the T4 stage. Next, we aimed to explore the associations between SCFAs and body weight change during pregnancy. We found that propionate was negatively correlated with body weight change (Figure 7D), while acetate was positively associated with body weight change (Figure 7E). Butyrate showed a negative correlation with body weight change, although the association was not statistically significant (Figure 7F). Finally, we aimed to identify the correlations among microbial biomarkers, SCFAs, and body weight change (Figure S4). We found that ASVs, including *Lachnospiraceae_XPB1014_group*, *Prevotellaceae_NK3B31_group*, and *Prevotellaceae_UCG_003*, were positively associated with body weight change. *unclassified_ Prevotellaceae* and *Prevotellaceae_UCG_003* were positively correlated with acetate.

## 4. Discussion

Although an increasing number of studies have examined how the composition and diversity of the pig fecal microbiome change with age, most of these investigations focus on either early life stages or commercial pigs bred for meat production [22,23,24]. In recent years, only a limited number of longitudinal studies have explored the dynamics of the gut microbiome in gravid hosts, including both pigs and humans [25]. Nevertheless, the results concerning the microbial composition of pregnant women remain inconsistent [26]. For instance, a study reported that the microbial communities from different body sites, including fecal samples, exhibited considerable stability throughout pregnancy [27], while another study demonstrated significant changes in the fecal microbiome during the course of gestation [28]. From pregnancy to weaning, dietary changes can significantly influence the composition of the gut microbiome. Monitoring microbial changes from pregnancy through weaning in humans is challenging due to numerous confounding factors, such as environmental influences and diet, which are difficult to control in human research.

The gut microbiota is predominantly composed of two major phyla, Firmicutes and Bacteroidetes, which collectively make up approximately 90% of the total microbial population [29]. The F/B ratio has been regarded as a pivotal index of gut microbiota health [30]. For example, a high F/B ratio has been repeatedly implicated in obesity and metabolic-related conditions [31]. In our study, we observed that the F/B ratio was highest at the T3 stage and significantly greater than the levels found at the T1 and T2 stages. Additionally, the fecal bacterial communities in sows displayed a time-driven pattern, characterized by a rapid shift in enterotypes from early pregnancy to the time of weaning. For example, the enterotype shifts from *Prevotellaceae_UCG_001* at the T1 stage to Bacteroides at the T3 stage. Interestingly, we found that the T2 stage was specifically enriched with a variety of ASVs belonging to *Prevotellaceae*, including *Prevotellaceae_NK3B31_group*, *Prevotella9*, *Prevotellaceae_UCG_003*, and unclassified *Prevotellaceae*. In this study, the sows’ dietary composition during gestation primarily consisted of corn (60%), bran (20%), and soybean meal (8%). As the pigs transitioned into the lactation stage, the diet was gradually adjusted to include corn (69%), soybean meal (14%), and expanded soybean (5%), reflecting the higher energy and protein demands of milk production and supporting the optimal body condition of sows. Bran is commonly regarded as a significant source of dietary fiber, and its inclusion in the diet of gestating sows could increase crude fiber intake, thereby promoting gastrointestinal motility and reducing the risk of constipation [32]. Sows experience significant fluctuations in hormone levels during the prepartum and postpartum periods. For example, previous studies have shown that estradiol (E2), a type of estrogen, has a concentration of approximately 75 pg/mL prepartum, which declines to about 5.5 pg/mL postpartum [33]. *Prevotella* spp. have been found to participate in the degradation of pregnenolone and progesterone, thereby promoting the biosynthesis of estrogens [34]. Research has also indicated a relationship between estradiol and increased appetite [35]. Therefore, we hypothesize that *Prevotella* spp. may enhance appetite in pregnant sows by promoting the production of estrogens such as estradiol, which in turn may contribute to increased gestational body weight gain and support fetal development.

The Bacteroides-driven enterotype is frequently observed in individuals with a high consumption of protein and animal fat, as is characteristic of a Western diet. On the other hand, individuals with diets rich in carbohydrates and fiber are more likely to have a *Prevotella*-dominant enterotype [36,37,38]. In line with human studies, we found that the absence of bran (crude fiber) in the feed during lactation drives a shift in enterotypes of sows from a *Prevotella_UCG_001*-dominated profile during pregnancy to a Bacteroides-dominated profile during lactation. Previous studies have demonstrated that dietary fiber serves as a crucial nutritional source for *Prevotella*, and a high intake of dietary fiber has been shown to enhance the abundance of *Prevotella* [39,40].

SCFAs are mainly generated in the large intestine through the fermentation of plant-derived carbohydrates by gut microbiota [41]. *Prevotella*, a group of gram-negative anaerobes, ferment dietary fiber to produce acetate [42]. In our study, we found that *Prevotella_UCG_003* and *unclassified_Prevotellaceae* were positively associated with acetate. In pregnant mice, acetate generated by the gut microbiota is capable of crossing the placenta and reducing postnatal allergic responses in offspring [43]. In addition, a positive correlation was observed between acetate levels and anthropometric measures (BMI) among overweight or obese pregnant women (prior to pregnancy) [15]. Studies have revealed that acetate plays a role in de novo lipogenesis and promotes hepatic cholesterogenesis, while Propionate has an inhibitory effect [44,45]. Thus, we conjecture that high maternal carriage of *Prevotella* taxa during pregnancy may be causally related to immune modulation and fat accumulation.

By performing a correlation analysis between differentially enriched KEGG pathways and microbial biomarkers at the T2 stage, we found that *Prevotella_9* was positively associated with arachidonic acid metabolism. A previous study revealed that arachidonic acid metabolism was activated in obesity-prone mice [46]. Earlier research demonstrated that arachidonic acid impacted obesity via the gut–hypothalamus–adipose–liver axis; i.e., arachidonic acid was shown to aggravate obesity, increase the abundance of pro-inflammatory microbiota, exacerbate nonalcoholic steatohepatitis, and trigger insulin resistance [47]. In addition, the genes involved in arachidonic acid metabolism were found in the genome of *Prevotella copri* isolates [26]. Interestingly, previous studies have shown that *Prevotella_9* may potentially represent *Prevotella copri* [48]. Therefore, we speculate that the increased abundance of *Prevotella_9* during pregnancy may promote fat deposition via arachidonic acid metabolism. However, this hypothesis requires further validation.

In addition to the enrichment of certain ASVs belonging to *Prevotella* at the T2 stage, we found that *Lachnospiraceae_XPB1014_group* was the most prominently enriched ASV at this stage. *Lachnospiraceae_XPB1014_group* is a genus-level taxonomic group within the family *Lachnospiraceae*. As is known, members of the *Lachnospiraceae* are vital bacteria involved in fiber degradation and SCFA production [49,50]. Recently, a study found that *Lachnospiraceae_XPB1014_group* was positively associated with metabolites belonging to unsaturated aliphatic hydrocarbons and fatty acid metabolism [51]. Interestingly, a 20% reduction in dietary protein levels led to an increase in the abundance of *Lachnospiraceae_XPB1014_group* at the genus level [52]. In our study, the lower crude protein content during pregnancy (T1 and T2) compared to the lactation stage (T3 and T4) suggests that the enrichment of *Lachnospiraceae_XPB1014_group* during pregnancy may contribute to fiber degradation and promote host health through the production of SCFAs. However, further exploration of the molecular interactions between *Lachnospiraceae_XPB1014_group* and host metabolism can provide a more comprehensive understanding of the underlying mechanisms.

At the T3 stage, we found that *Eubacterium_coprostanoligenes_group* was significantly enriched. *Eubacterium_coprostanoligenes_group* contains the gene responsible for encoding IsmA, a crucial enzyme in cholesterol metabolism [53]. Working synergistically with *Oscillibacter*, it transforms cholesterol into coprostanol, a compound that is non-absorbable by the intestine, thereby lowering blood cholesterol levels and influencing host fat metabolism [54,55]. Earlier studies have demonstrated that *Eubacterium_coprostanoligenes_group* is inversely correlated with triglycerides, total cholesterol, and low-density lipoprotein cholesterol (LDL-C) [56,57]. Moreover, as a butyrate-producing bacterium, *Eubacterium_coprostanoligenes_group* may play a role in maintaining colonic tissue integrity, supporting a healthy mucosal layer, and mitigating intestinal inflammation [58]. We hypothesize that *Eubacterium_coprostanoligenes_group* may contribute to the recovery of postpartum body condition and the alleviation of intestinal inflammation in sows.

*Lachnospiraceae_NK4A136_group* is a member of the *Lachnospiraceae* family. In our study, we observed that it was significantly enriched in the T4 stage. Previous research has demonstrated that *Lachnospiraceae_NK4A136_group* is involved in the production of SCFAs, including acetic acid and butyric acid [59]. Additionally, *Lachnospiraceae_NK4A136_group* may exhibit anti-inflammatory properties [60], and its relative abundance was found to be reduced in mice with immune dysfunction induced by cyclophosphamide [61]. Furthermore, another study reported a negative correlation between *Lachnospiraceae_NK4A136_group* and obesity [52]. At the weaning stage, sows often exhibit a marked decline in body conditions (e.g., body weight and backfat), largely due to the physiological demands of lactation, fluctuations in feed consumption, and inflammatory processes [62]. Therefore, we speculate that the significant enrichment of *Lachnospiraceae_NK4A136_group* in the weaning stage may help reduce systemic inflammation and prevent excessive obesity in sows, thus favorably influencing postpartum estrus. Lastly, our study was primarily limited by the absence of metagenomic data, which restricted the further identification of differential microbial species. Future research should investigate the impacts of key bacterial species on maternal livestock during both pregnancy and postpartum stages using culturomics and animal experiments.

## 5. Conclusions

In this study, we tracked the dynamic changes in the gut microbiota and fecal SCFAs of sows from pregnancy to the weaning stages. We found that the gut microbiota of sows during pregnancy was predominantly composed of *Prevotella*, and several ASVs belonging to the family *Prevotellaceae* were associated with arachidonic acid metabolism and fecal acetate production. In the postpartum period, *Lachnospiraceae_NK4A136_group* and *Eubacterium_coprostanoligenes_group* were the dominant bacterial taxa. Notably, fecal propionate levels were negatively correlated with body weight change during pregnancy, whereas acetate levels showed a positive correlation. Future studies should investigate whether and how the dietary inclusion of alternative fiber sources, such as inulin, sugar beet pulp, and alfalfa meal, can exert beneficial effects on pregnant sows through improvements in gut microbiota. Moreover, the integration of culturomics and multi-omics approaches is required to elucidate the functional roles of specific microbial strains belonging to *Prevotella*, *Lachnospiraceae_NK4A136_group*, and *Eubacterium_coprostanoligenes_group*. Taken together, these results provide a foundation for further understanding the potential roles of gut microbiota and SCFAs in maternal health throughout the gestation and postpartum periods in sows.

## Data Availability

The data presented in this study are available on request from the corresponding author.

## Supplementary Materials

The following supporting information can be downloaded at: https://www.mdpi.com/article/10.3390/ani15152209/s1, Table S1. Nutrient components of commercial formula feed provided to Yorkshire sows. Table S2. Summary description of 16S rRNA gene sequencing data. Table S3. Comparison of Shannon index among four stages. Figure S1. Comparison of raw read counts among the four stages from pregnancy to weaning (n = 25/per stage). One-way ANOVA. *** *p* < 0.001. Figure S2. Optimal number of clusters among four stages from pregnancy to weaning. CH index: Calinski–Harabasz index. Figure S3. Interactions among bacteria. (A) The microbiota interaction networks at the T2 (A) and T4 (B) stages based on the following criteria: |r| > 0.7 and FDR < 0.001. The pink and blue lines indicate positive and negative correlations, respectively. Figure S4. Correlation analysis among microbial biomarkers, SCFAs, and body weight change during pregnancy. Spearman’s rank correlation analysis. * *p* < 0.05, ** *p* < 0.01, *** *p* < 0.001.

## References

[1] Tian M., Li Q., Zheng T., Yang S., Chen F., Guan W., Zhang S. (2023). Maternal microbe-specific modulation of the offspring microbiome and development during pregnancy and lactation. Gut Microbes.

[2] Hassib L., de Oliveira C.L., Rouvier G.A., Kanashiro A., Guimarães F.S., Ferreira F.R. (2023). Maternal microbiome disturbance induces deficits in the offspring’s behaviors: A systematic review and meta-analysis. Gut Microbes.

[3] Qu R., Zhang Y., Ma Y., Zhou X., Sun L., Jiang C., Zhang Z., Fu W. (2023). Role of the Gut Microbiota and Its Metabolites in Tumorigenesis or Development of Colorectal Cancer. Adv. Sci..

[4] Amato K.R., Pradhan P., Mallott E.K., Shirola W., Lu A. (2024). Host-gut microbiota interactions during pregnancy. Evol. Med. Public Health.

[5] Koren O., Konnikova L., Brodin P., Mysorekar I.U., Collado M.C. (2024). The maternal gut microbiome in pregnancy: Implications for the developing immune system. Nat. Rev. Gastroenterol. Hepatol..

[6] Hu J., Chen J., Xu X., Hou Q., Ren J., Yan X. (2023). Correction: Gut microbiota-derived 3-phenylpropionic acid promotes intestinal epithelial barrier function via AhR signaling. Microbiome.

[7] Lan Q., Liufu S., Liu X., Ai N., Xu X., Li X., Yu Z., Yin Y., Liu M., Ma H. (2023). Comprehensive analysis of transcriptomic and metabolomic profiles uncovered the age-induced dynamic development pattern of subcutaneous fat in Ningxiang pig. Gene.

[8] Bordbar F., Mohammadabadi M., Jensen J., Xu L., Li J., Zhang L. (2022). Identification of Candidate Genes Regulating Carcass Depth and Hind Leg Circumference in Simmental Beef Cattle Using Illumina Bovine Beadchip and Next-Generation Sequencing Analyses. Animals.

[9] Zhu B., Wang X., Li L. (2010). Human gut microbiome: The second genome of human body. Protein Cell.

[10] Smith P.M., Howitt M.R., Panikov N., Michaud M., Gallini C.A., Bohlooly Y.M., Glickman J.N., Garrett W.S. (2013). The microbial metabolites, short-chain fatty acids, regulate colonic Treg cell homeostasis. Science.

[11] Bergman E.N. (1990). Energy contributions of volatile fatty acids from the gastrointestinal tract in various species. Physiol. Rev..

[12] Ganal-Vonarburg S.C., Hornef M.W., Macpherson A.J. (2020). Microbial-host molecular exchange and its functional consequences in early mammalian life. Science.

[13] Priyadarshini M., Thomas A., Reisetter A.C., Scholtens D.M., Wolever T.M., Josefson J.L., Layden B.T. (2014). Maternal short-chain fatty acids are associated with metabolic parameters in mothers and newborns. Transl. Res..

[14] Zavalza-Gómez A.B., Anaya-Prado R., Rincón-Sánchez A.R., Mora-Martínez J.M. (2008). Adipokines and insulin resistance during pregnancy. Diabetes Res. Clin. Pract..

[15] Szczuko M., Kikut J., Maciejewska D., Kulpa D., Celewicz Z., Ziętek M. (2020). The Associations of SCFA with Anthropometric Parameters and Carbohydrate Metabolism in Pregnant Women. Int. J. Mol. Sci..

[16] Chen S., Li J., Ren S., Gao Y., Zhou Y., Xuan R. (2022). Expression and clinical significance of short-chain fatty acids in pregnancy complications. Front. Cell. Infect. Microbiol..

[17] Hou N., Du X., Wu S. (2022). Advances in pig models of human diseases. Anim. Models Exp. Med..

[18] Liang C., Tseng H.C., Chen H.M., Wang W.C., Chiu C.M., Chang J.Y., Lu K.Y., Weng S.L., Chang T.H., Chang C.H. (2017). Diversity and enterotype in gut bacterial community of adults in Taiwan. BMC Genom..

[19] Arumugam M., Raes J., Pelletier E., Le Paslier D., Yamada T., Mende D.R., Fernandes G.R., Tap J., Bruls T., Batto J.M. (2011). Enterotypes of the human gut microbiome. Nature.

[20] Kumar L., Futschik M.E. (2007). Mfuzz: A software package for soft clustering of microarray data. Bioinformation.

[21] Lan Q., Lian Y., Peng P., Yang L., Zhao H., Huang P., Ma H., Wei H., Yin Y., Liu M. (2023). Association of gut microbiota and SCFAs with finishing weight of Diannan small ear pigs. Front. Microbiol..

[22] Jurburg S.D., Bossers A. (2021). Age Matters: Community Assembly in the Pig Fecal Microbiome in the First Month of Life. Front. Microbiol..

[23] Chen Q., Zhang X., Shi W., Du X., Ma L., Wang W., Tao S., Xiao Y. (2023). Longitudinal Investigation of Enteric Virome Signatures from Parental-Generation to Offspring Pigs. Microbiol. Spectr..

[24] Gaire T.N., Scott H.M., Noyes N.R., Ericsson A.C., Tokach M.D., William H., Menegat M.B., Vinasco J., Nagaraja T.G., Volkova V.V. (2024). Temporal dynamics of the fecal microbiome in female pigs from early life through estrus, parturition, and weaning of the first litter of piglets. Anim. Microbiome.

[25] Chen Z., Yang H., Fu H., Wu L., Liu M., Jiang H., Liu Q., Wang Y., Xiong S., Zhou M. (2022). Gut bacterial species in late trimester of pregnant sows influence the occurrence of stillborn piglet through pro-inflammation response. Front. Immunol..

[26] Chen C., Fang S., Wei H., He M., Fu H., Xiong X., Zhou Y., Wu J., Gao J., Yang H. (2021). Prevotella copri increases fat accumulation in pigs fed with formula diets. Microbiome.

[27] DiGiulio D.B., Callahan B.J., McMurdie P.J., Costello E.K., Lyell D.J., Robaczewska A., Sun C.L., Goltsman D.S., Wong R.J., Shaw G. (2015). Temporal and spatial variation of the human microbiota during pregnancy. Proc. Natl. Acad. Sci. USA.

[28] Koren O., Goodrich J.K., Cullender T.C., Spor A., Laitinen K., Bäckhed H.K., Gonzalez A., Werner J.J., Angenent L.T., Knight R. (2012). Host remodeling of the gut microbiome and metabolic changes during pregnancy. Cell.

[29] The Human Microbiome Project Consortium (2012). Structure, function and diversity of the healthy human microbiome. Nature.

[30] Li W., Ma Z.S. (2020). FBA Ecological Guild: Trio of Firmicutes-Bacteroidetes Alliance against Actinobacteria in Human Oral Microbiome. Sci. Rep..

[31] Woting A., Blaut M. (2016). The Intestinal Microbiota in Metabolic Disease. Nutrients.

[32] Dumniem N., Boonprakob R., Panvichitra C., Thongmark S., Laohanarathip N., Parnitvoraphoom T., Changduangjit S., Boonmakaew T., Teshanukroh N., Tummaruk P. (2024). Impacts of Fiber Supplementation in Sows during the Transition Period on Constipation, Farrowing Duration, Colostrum Production, and Pre-Weaning Piglet Mortality in the Free-Farrowing System. Animals.

[33] Molokwu E.C.I., Wagner W.C. (1973). Endocrine physiology of the puerperal sow. J. Anim. Sci..

[34] Liu M., Zhang J., Zhou Y., Xiong S., Zhou M., Wu L., Liu Q., Chen Z., Jiang H., Yang J. (2023). Gut microbiota affects the estrus return of sows by regulating the metabolism of sex steroid hormones. J. Anim. Sci. Biotechnol..

[35] Vigil P., Meléndez J., Petkovic G., Del Río J.P. (2022). The importance of estradiol for body weight regulation in women. Front. Endocrinol..

[36] Wu G.D., Chen J., Hoffmann C., Bittinger K., Chen Y.Y., Keilbaugh S.A., Bewtra M., Knights D., Walters W.A., Knight R. (2011). Linking long-term dietary patterns with gut microbial enterotypes. Science.

[37] Lim M.Y., Rho M., Song Y.M., Lee K., Sung J., Ko G. (2014). Stability of gut enterotypes in Korean monozygotic twins and their association with biomarkers and diet. Sci. Rep..

[38] De Moraes A.C., Fernandes G.R., da Silva I.T., Almeida-Pititto B., Gomes E.P., Pereira A.D., Ferreira S.R. (2017). Enterotype May Drive the Dietary-Associated Cardiometabolic Risk Factors. Front. Cell. Infect. Microbiol..

[39] De Filippo C., Cavalieri D., Di Paola M., Ramazzotti M., Poullet J.B., Massart S., Collini S., Pieraccini G., Lionetti P. (2010). Impact of diet in shaping gut microbiota revealed by a comparative study in children from Europe and rural Africa. Proc. Natl. Acad. Sci. USA.

[40] Chung W.S.F., Walker A.W., Bosscher D., Garcia-Campayo V., Wagner J., Parkhill J., Duncan S.H., Flint H.J. (2020). Relative abundance of the Prevotella genus within the human gut microbiota of elderly volunteers determines the inter-individual responses to dietary supplementation with wheat bran arabinoxylan-oligosaccharides. BMC Microbiol..

[41] Feng W., Ao H., Peng C. (2018). Gut Microbiota, Short-Chain Fatty Acids, and Herbal Medicines. Front. Pharmacol..

[42] Franke T., Deppenmeier U. (2018). Physiology and central carbon metabolism of the gut bacterium Prevotella copri. Mol. Microbiol..

[43] Thorburn A.N., McKenzie C.I., Shen S., Stanley D., Macia L., Mason L.J., Roberts L.K., Wong C.H., Shim R., Robert R. (2015). Evidence that asthma is a developmental origin disease influenced by maternal diet and bacterial metabolites. Nat. Commun..

[44] Wong J.M., de Souza R., Kendall C.W., Emam A., Jenkins D.J. (2006). Colonic health: Fermentation and short chain fatty acids. J. Clin. Gastroenterol..

[45] Weitkunat K., Schumann S., Nickel D., Kappo K.A., Petzke K.J., Kipp A.P., Blaut M., Klaus S. (2016). Importance of propionate for the repression of hepatic lipogenesis and improvement of insulin sensitivity in high-fat diet-induced obesity. Mol. Nutr. Food Res..

[46] Zhang H., Chen S., Yang L., Zhang S., Qin L., Jiang H. (2024). Distinct Gut Microbiota and Arachidonic Acid Metabolism in Obesity-Prone and Obesity-Resistant Mice with a High-Fat Diet. Nutrients.

[47] Zhuang P., Shou Q., Lu Y., Wang G., Qiu J., Wang J., He L., Chen J., Jiao J., Zhang Y. (2017). Arachidonic acid sex-dependently affects obesity through linking gut microbiota-driven inflammation to hypothalamus-adipose-liver axis. Biochim. Et Biophys. Acta (BBA)-Mol. Basis Dis..

[48] Wells P.M., Adebayo A.S., Bowyer R.C.E., Freidin M.B., Finckh A., Strowig T., Lesker T.R., Alpizar-Rodriguez D., Gilbert B., Kirkham B. (2020). Associations between gut microbiota and genetic risk for rheumatoid arthritis in the absence of disease: A cross-sectional study. Lancet Rheumatol..

[49] Li D., Wang P., Wang P., Hu X., Chen F. (2018). Gut microbiota promotes production of aromatic metabolites through degradation of barley leaf fiber. J. Nutr. Biochem..

[50] Telle-Hansen V.H., Gaundal L., Bastani N., Rud I., Byfuglien M.G., Gjøvaag T., Retterstøl K., Holven K.B., Ulven S.M., Myhrstad M.C.W. (2022). Replacing saturated fatty acids with polyunsaturated fatty acids increases the abundance of Lachnospiraceae and is associated with reduced total cholesterol levels—A randomized controlled trial in healthy individuals. Lipids Health Dis..

[51] Su J., Li J., Azad M.A.K., Wang W., Luo Z., Wang J., Yin J., Yin Y., Tan B., Chen J. (2024). Dynamic distribution of gut microbiota-metabolites during post-weaning longissimus dorsi muscle development in Ningxiang pigs. Microbiol. Spectr..

[52] Zheng J., Duan Y., Zheng C., Yu J., Li F., Guo Q., Yin Y. (2022). Long-Term Protein Restriction Modulates Lipid Metabolism in White Adipose Tissues and Alters Colonic Microbiota of Shaziling Pigs. Animals.

[53] Wu Y., Zhai S., Fang M., Zhang H., Chen Y. (2024). Evaluation of the growth performance, meat quality, and gut microbiota of broilers fed diets containing walnut green husk extract. Poult. Sci..

[54] Li C., Stražar M., Mohamed A.M.T., Pacheco J.A., Walker R.L., Lebar T., Zhao S., Lockart J., Dame A., Thurimella K. (2024). Gut microbiome and metabolome profiling in Framingham heart study reveals cholesterol-metabolizing bacteria. Cell.

[55] Freier T.A., Beitz D.C., Li L., Hartman P.A. (1994). Characterization of Eubacterium coprostanoligenes sp. nov., a cholesterol-reducing anaerobe. Int. J. Syst. Evol. Microbiol..

[56] Wei W., Jiang W., Tian Z., Wu H., Ning H., Yan G., Zhang Z., Li Z., Dong F., Sun Y. (2021). Fecal g. Streptococcus and g. Eubacterium_coprostanoligenes_group combined with sphingosine to modulate the serum dyslipidemia in high-fat diet mice. Clin. Nutr..

[57] Deng M., Zhang S., Dong L., Huang F., Jia X., Su D., Chi J., Muhammad Z., Ma Q., Zhao D. (2022). Shatianyu (*Citrus grandis* L. Osbeck) Flavonoids and Dietary Fiber in Combination Are More Effective Than Individually in Alleviating High-Fat-Diet-Induced Hyperlipidemia in Mice by Altering Gut Microbiota. J. Agric. Food Chem..

[58] Fang Y., Zhang Y., Liu Q., Zheng Z., Ren C., Zhang X. (2024). Assessing the causal relationship between gut microbiota and diabetic nephropathy: Insights from two-sample Mendelian randomization. Front. Endocrinol..

[59] Li H., Liu F., Lu J., Shi J., Guan J., Yan F., Li B., Huo G. (2020). Probiotic Mixture of Lactobacillus plantarum Strains Improves Lipid Metabolism and Gut Microbiota Structure in High Fat Diet-Fed Mice. Front. Microbiol..

[60] Li J., Wu T., Li N., Wang X., Chen G., Lyu X. (2019). Bilberry anthocyanin extract promotes intestinal barrier function and inhibits digestive enzyme activity by regulating the gut microbiota in aging rats. Food Funct..

[61] Zhu G., Jiang Y., Yao Y., Wu N., Luo J., Hu M., Tu Y., Xu M. (2019). Ovotransferrin ameliorates the dysbiosis of immunomodulatory function and intestinal microbiota induced by cyclophosphamide. Food Funct..

[62] Song B., Yu L., Liu X., Goswami N., Gong R., Ren Z. (2024). Exploring optimal folic acid supplementation levels for lactating-pregnant rabbit does with different litter size. J. Anim. Sci..

