# Supplementary material for: Temporal Dynamics of Fecal Microbiome and Short-Chain Fatty Acids in Sows from Early Pregnancy to Weaning
Source: Animals (Basel). 2025 Jul 27;15(15):2209. doi: 10.3390/ani15152209 (PMC12345544; doi:10.3390/ani15152209)
Supplement: Supplementary file 1 [file animals-15-02209-s001.zip › animals-3754700-supplementary Figure.pdf]

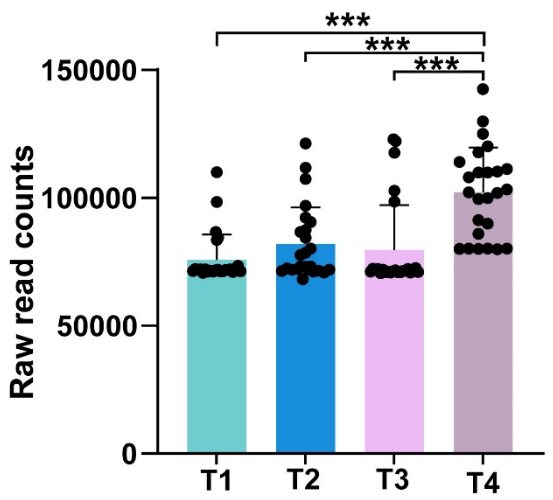

Figure S1. Comparison of raw read counts among the four stages from pregnancy to weaning (n=25/per stage). One-way ANOVA. *** p < 0.001.

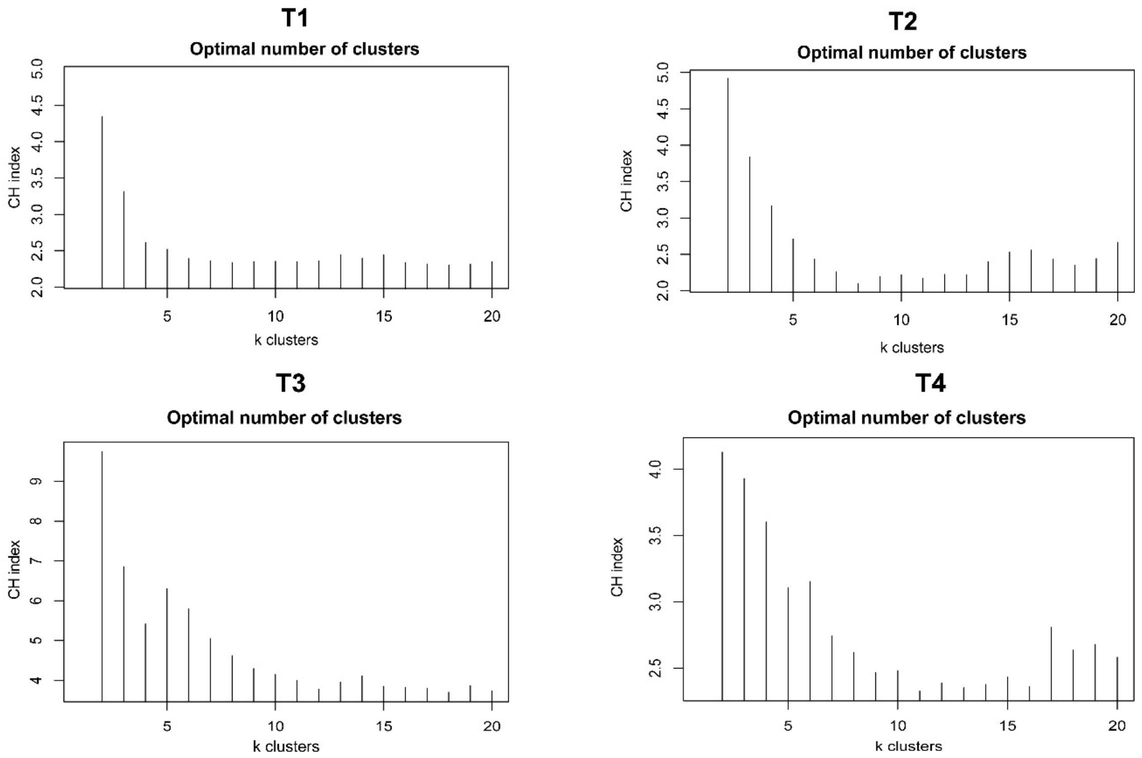

Figure S2. Optimal number of clusters among the four stages from pregnancy to weaning. CH index: Calinski_Harabasz index.

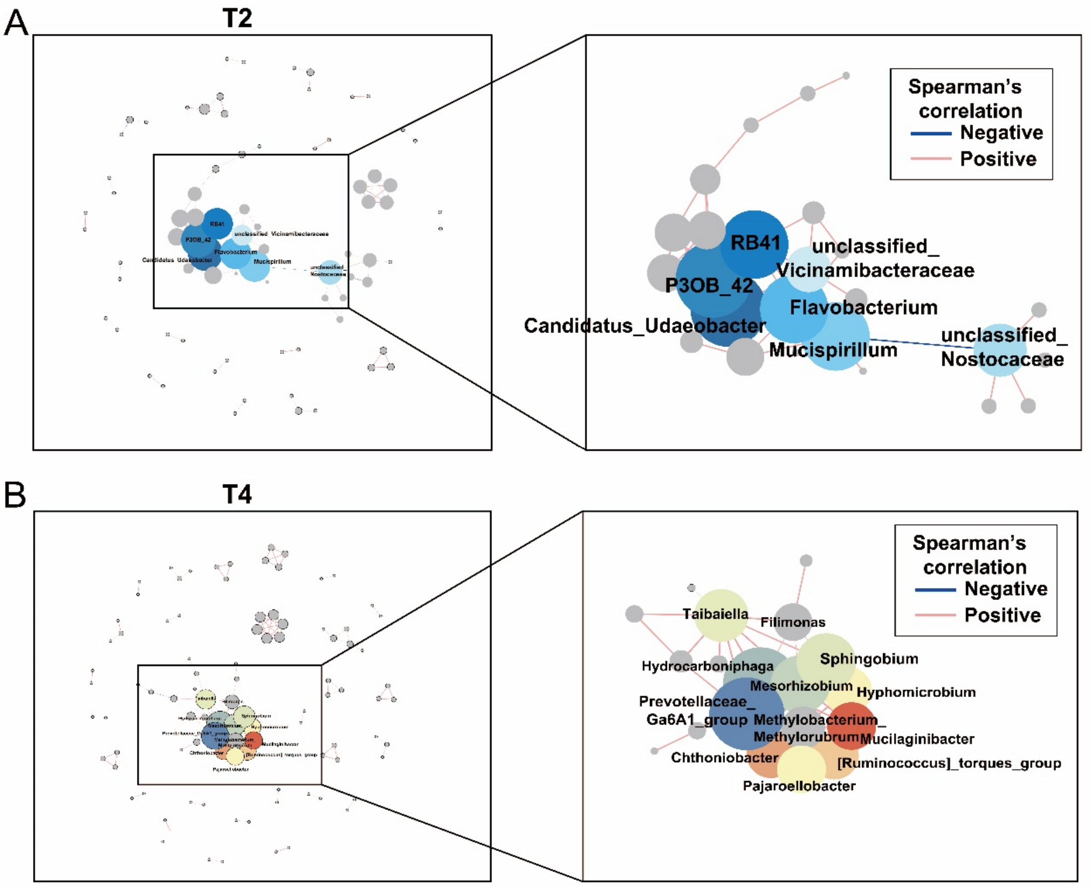

Figure S3. Interactions among bacteria. (A) The microbiota interaction networks at the T2 (A) and T4 (B) stages based on the following criteria: $|r| > 0.7$ and FDR < 0.001. The Pink and blue lines indicate positive and negative correlations, respectively.

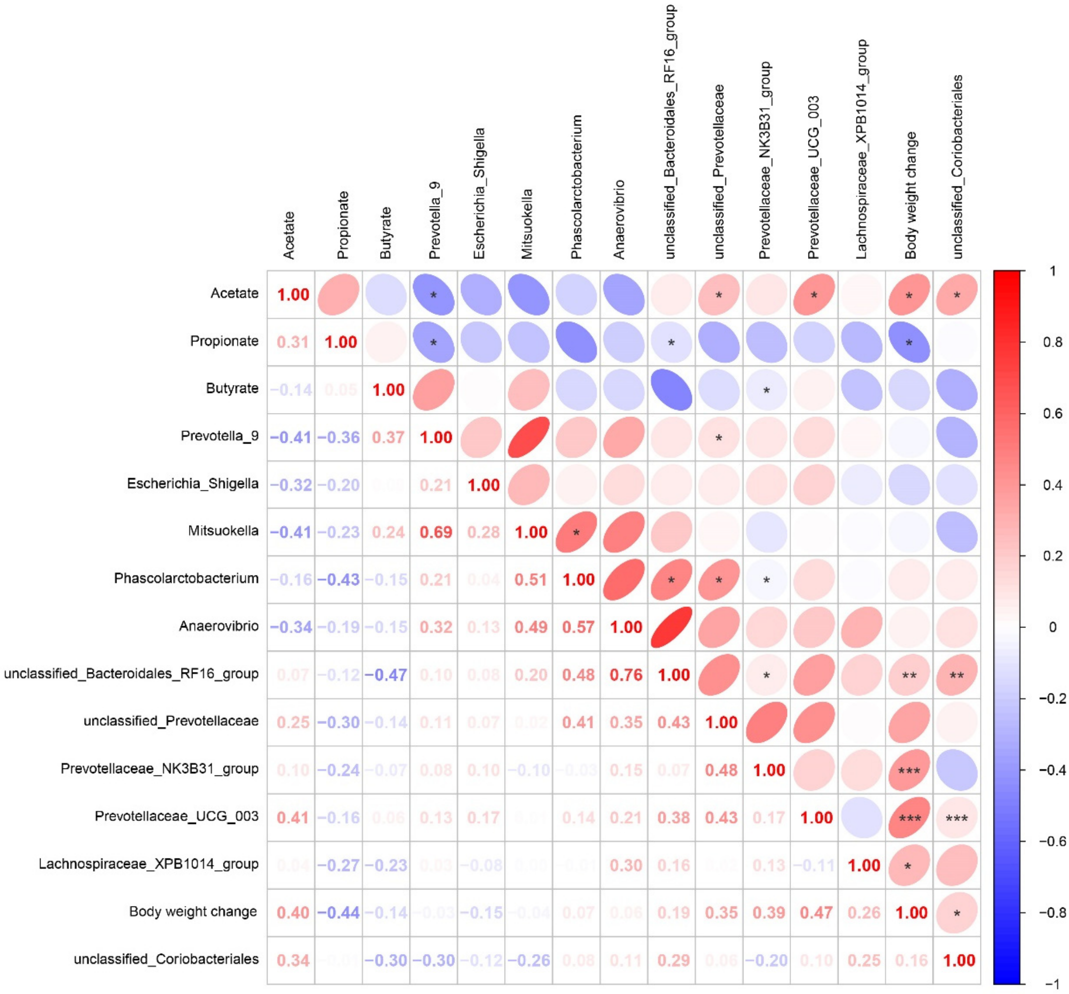

Figure S4. Correlation analysis among microbial biomarkers, SCFAs, and body weight change during pregnancy. Spearman's rank correlation analysis. * $p < 0.05$, ** $p < 0.01$, *** $p < 0.001$.
